# Cas11 augments Cascade functions in type I-E CRISPR system but is redundant for gene silencing and plasmid interference

**DOI:** 10.1042/BCJ20253056

**Published:** 2025-06-11

**Authors:** Neha Pandey, Chitra Seetharam Misra, Devashish Rath

**Affiliations:** 1 Applied Genomics Section, Bio-Science Group, Bhabha Atomic Research Centre, Mumbai, India; 2 Life Sciences, Mumbai University, Vidya Nagari, Kalina, Santacruz East, Mumbai, India; 3 Homi Bhabha National Institute, Anushaktinagar, Mumbai, India

**Keywords:** Cas11, Cascade, CRISPR, *Escherichia coli*, gene silencing, type I-E

## Abstract

The structural and mechanistic complexity of *Escherichia coli’*s type I Clustered Regularly Interspaced Short Palindromic Repeats/CRISPR-associated (CRISPR-Cas) system, compared with the multidomain, single effector protein-based type II systems, limits its application in genome editing and silencing. Despite the higher prevalence of the type I endogenous systems in bacteria, significant research has focused on improving the type II systems. While the type-I CRISPR system possesses several advantages over others, it may benefit from further studies to simplify the system for ease of use. To enable this, the dispensability of the type-I Cascade components (Cas8, Cas11, Cas7, Cas5 and Cas6) for genome editing and silencing applications was evaluated *in vivo*. We created deletion variants of each of the Cascade components and investigated their effects on gene silencing and plasmid interference in two genetically distinct *E. coli* lineages: BW25113, a K-12 strain that bears an endogenous, albeit repressed type I-E CRISPR system; and BL21, a natural mutant lacking the type I-E CRISPR-Cas system. Cas8, Cas7 and Cas5 were found to be indispensable for gene silencing and plasmid interference. Dispensability of Cas6, which is involved in crRNA maturation, was strain-dependent. Notably, Cas11, which has no definitive function assigned to it, was found to be dispensable for gene silencing and plasmid interference.

## Introduction

The Clustered Regularly Interspaced Short Palindromic Repeats/CRISPR-associated (CRISPR-Cas) systems are adaptive immune mechanisms used by bacteria and archaea to defend against invasive nucleic acids such as bacteriophages and plasmids [1]. These systems rely on signature Cas proteins along with a functional CRISPR RNA (crRNA) to mediate precise target recognition and its nucleolytic degradation. CRISPR/Cas systems are categorized into two major classes with several types and sub-types within each class. The class 2 systems have a single Cas effector protein, while class 1 systems typically have a multi-Cas protein effector complex. The types and sub-types are grouped based on their mode of action and the presence of signature Cas proteins [2]. Among the six types (I-VI), type I, type II and type III systems, characterized by their distinctive signature proteins: Cas3, Cas9 and Cas10, respectively, were discovered early and were extensively studied [1]. The type II CRISPR/Cas system, due to its relative structural simplicity, has been particularly well understood and is widely used for genome-editing applications [3].

**Figure 1:**
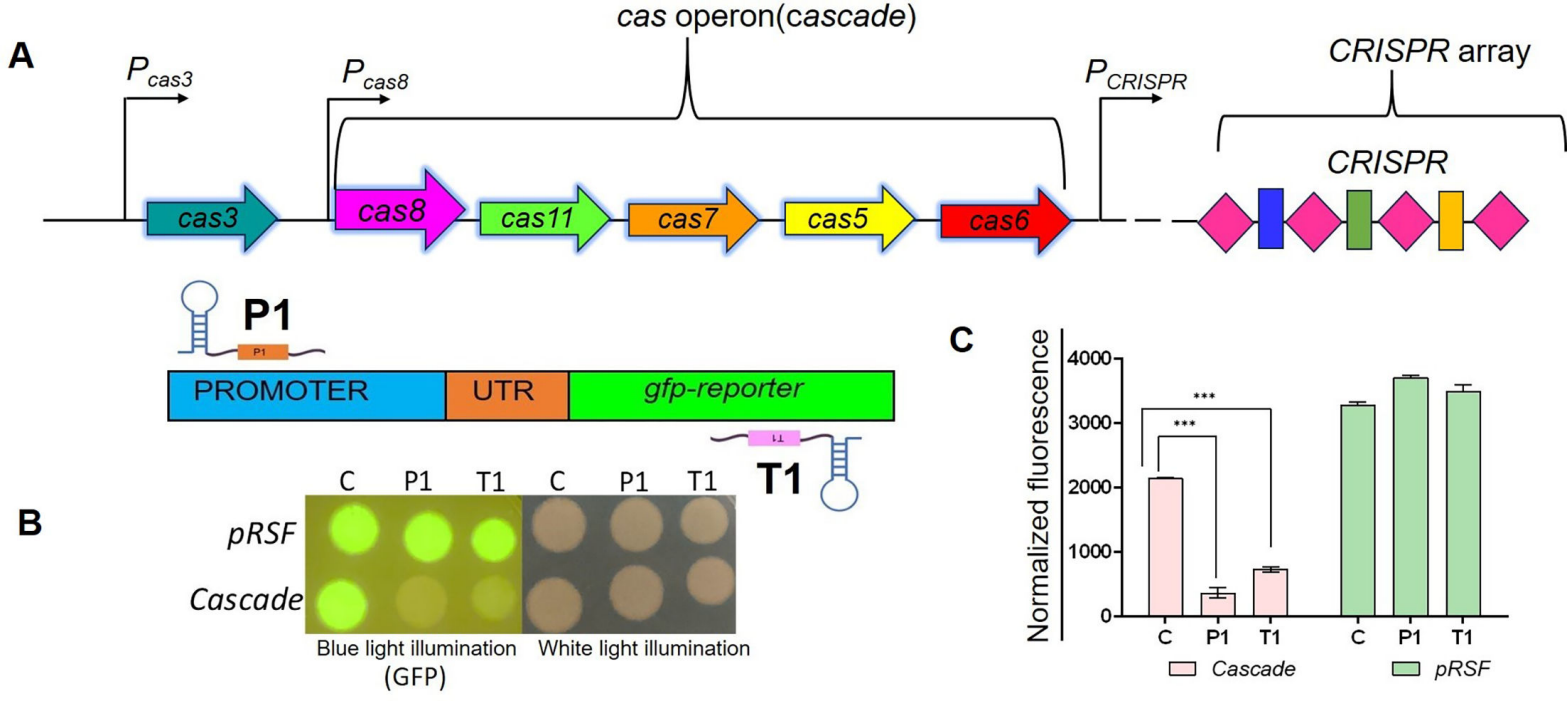
**. Assessment of the contribution of background expression of the Cascade system to *gfp* silencing in *E. coli* MLS367**. Schematic representation of the Cascade operon in *E. coli* MLS367 (**A**). Fluorescence was monitored in MLS367 cells expressing *gfp* and crRNA (targeting *gfp* promoter, P1 or ORF, T1 or a non-targeting scrambled control, C) and carrying a plasmid-encoding Cascade operon or empty vector (pRSF), through spot assays (**B**) and in broth cultures at 6 h post-induction (**C**). Spot data are representative of experiments repeated three times (**B**). Data plotted is mean ± SEM of three independent experiments (**C**) ***P < 0.001 by *t* test.

The type I CRISPR/Cas system features a multiprotein complex known as Cascade (CRISPR-associated complex for antiviral defense) and a distinct nuclease, Cas3, that is responsible for the nucleolytic degradation of the target nucleic acid [4]. Most of the knowledge of type I systems comes from studies of the type I-E system in *Escherichia coli* (*E. coli* (Figure 1A). The interaction of Cascade with foreign DNA begins with a search for a sequence called a protospacer-adjacent motif (PAM) [5]. PAM recognition is followed by crRNA-guided search for complementarity between the crRNA spacer and the DNA sequence [6,7]. The binding of the spacer with the target triggers a conformational change that recruits Cas3. Cas3 recruitment by Cascade leads to degradation of the non-target strand in the 3′–5′ direction [4]. The structure of the surveillance complex formed by Cascade has been solved [8-10]. The type I-E Cascade complex with crRNA (crRNP) consists of five Cas proteins (Cas8, Cas11, Cas7, Cas5 and Cas6) and a 61-nucleotide (nt) mature crRNA. The horseshoe-shaped structure of this complex has a molecular mass of 405 kDa, comprising one subunit of Cas8 (earlier Cse1), two subunits of Cas11 (earlier Cse2), six subunits of Cas7 and one subunit each of Cas5 and Cas6 (earlier Cas6e). The 61-nt crRNA is a highly structured molecule, which is divided into a 32-nt spacer, an 8-nt-long handle at the 5′-end, followed by a 21-nt-long repeat sequence with a terminal hairpin at the 3′-end. *In vitro* studies have shown that the RNA endonuclease Cas6, situated at one end of the Cascade structure, converts pre-crRNA into mature crRNA, interacts with the downstream repeat-derived 3' handle of the crRNA and remains associated with the rest of the complex for further action [11,12]. The hexameric helical structure of Cas7 protects the crRNA, while Cas8 is responsible for PAM recognition and recruitment of Cas3. A dimer of two small subunits, Cas11, is thought to stabilize the formation of an R-loop structure and bind the displaced DNA strand [10]. The crystal structure reveals the roles of each Cascade component in both annealing to the target site and maintaining structural stability.

The type I-E system from *E. coli* has been exploited for several applications. Cascade, along with Cas3, has been used for targeted DNA degradation, for sequence-specific killing of microbes, and for genome editing [13,14]. In the absence of Cas3, Cascade stably binds to target DNA without cleaving it, a property that has been used for programmable gene silencing. Engineering of crRNA to direct binding of Cascade to a promoter demonstrated efficient gene silencing (up to ∼1000-fold), while binding of the Cascade to within ORF led to a more modest silencing of the gene [15,16]. This has provided a very powerful tool to study essential genes in *E. coli* and other bacteria [17-19]. The type-I CRISPR system may offer several advantages over the type-II and type-V systems [10]. Type-I Cascade recognizes a longer stretch of DNA (~32 bases) compared with other CRISPR systems (typically ~20 bases), which is likely to enhance the specificity of targeting. In addition, the processing of pre-RNA by Cas6 enables easier design of multiple targets. The multi-subunit protein complex also allows easy functionalization and programming for varied applications. Lastly, Cas3, being a processive enzyme, offers the possibility of creating larger deletions around the targeted locus [20]. A minimal Cascade with lesser number of Cas proteins is likely to enhance the applicability of this system and make it easier to adapt it in heterologous systems. Here, towards the objective of constructing a minimal Cascade, a study was undertaken to decipher the dispensability of its components for gene silencing and plasmid interference in *E. coli*. We created different deletion variants of all five Cascade components and assessed their functionality in terms of gene silencing and plasmid interference in two different *E. coli* genetic backgrounds. Our results show that Cas8, Cas5, Cas7 and Cas6 are indispensable for gene silencing and plasmid interference; however, an engineered Cascade complex without Cas11 shows gene silencing and plasmid interference, indicating functional redundancy.

## Results

### Deletion of individual Cascade components alters the expression of other components from the Cascade operon

The regions containing CRISPR-Cas loci are the fastest evolving regions in the genome of bacteria [21]. Furthermore, horizontal gene transfer contributes to the rapid evolution of these loci. In order to confirm the status of CRISPR-Cas loci in our laboratory strains, PCR amplification of *cas3* and Cascade operon, which are key components of type I-E CRISPR-Cas system, was carried out. Specific primers for the Cascade operon and *cas3* were used for amplification. No PCR products were observed for both *cas3* and Cascade operon in *E. coli* BL21 (DE3), confirming their absence in this strain (Supplementary Figure S1). While *E. coli* K-12 strain BW25113 produced PCR products of expected sizes, 4.2 kb for *cascade* and 2.6 kb for *cas3*, MLS367, a Δ*cas3* derivative of BW25113, showed amplification of the Cascade operon but not for *cas3* (Supplementary Figure S1). Previous reports indicate that the Cascade operon is repressed in BW25113 [22,23]. We assessed whether residual expression of Cascade can support gene silencing. To test this, plasmids pZE12Luc encoding crRNA targeting the promoter (P1) or the ORF (T1) of *gfp* or a non-targeting scrambled crRNA, and pEH9 plasmid harboring a Venus-*gfp* gene downstream of a PLtetO-1 promoter were transformed into MLS367 [16]. Introduction of Cascade (pRS-Cas) in cells containing *gfp* targeting crRNA led to about a seven-fold decrease in GFP fluorescence compared with non-target control (Figure 1B and C). However, upon introduction of the empty vector pRSF1b, GFP fluorescence levels were similar to those obtained with the non-target control (Figure 1B and C). The results show that in the absence of the expression of Cascade from an external plasmid, no *gfp* silencing is observed in this strain, indicating that the chromosomal Cascade operon is repressed and is unable to support gene silencing in the presence of targeting crRNA.

The genes coding for all five components of Cascade, Cas8, Cas11, Cas7, Cas5 and Cas6, are transcribed from a single operon in the type I-E system of *E. coli*. Using pRS-Cas as a template, each of the five components of the Cascade operon was deleted separately. The expression of Cascade subunits in the deletion variants generated was checked by the separation of total proteins on SDS-PAGE followed by Coomassie Brilliant Blue staining. *E. coli* BL21 (DE3) was individually transformed with pRS-Cas, pRS-Δcas6, pRS-Δcas5, pRS-Δcas7, pRS-Δcas11 and pRS-Δcas8, and the cells were induced with 0.5 mM IPTG (Isopropyl β-D-thiogalactopyranoside). Total protein from these cells was analyzed along with an uninduced control on 13% SDS-PAGE (Supplementary Figure S2). In cells harboring pRS-Cas, the expression of Cas5 (25 kDa) and Cas6 (23 kDa) was not discernible, likely due to the polar effect within the operon. In cells expressing full-length Cascade (pRS-Cas) or pRS-Δcas6 or pRS-Δcas5, the expression of Cas8, Cas11 and Cas7 was apparent as protein bands of sizes 55 kDa, 17 kDa and 40 kDa, respectively. Deletion of *cas8* in plasmid-borne Cascade operon made the expression of Cas6 more apparent, in addition to Cas11 and Cas7, albeit at lower levels. In case of *cas11* deletion, all four remaining subunits were expressed, with Cas8 and Cas7 showing higher levels in comparison with Cas6. Overall, the analysis showed that Cas5 and Cas6 are expressed at low levels from the Cascade operon, with the expression of the former being hard to discern in our assay (Supplementary Figure S2). The expression of downstream subunits improved with the deletion of a preceding gene in the operon.

### Essentiality of Cascade components for gene silencing

**Figure 2:**
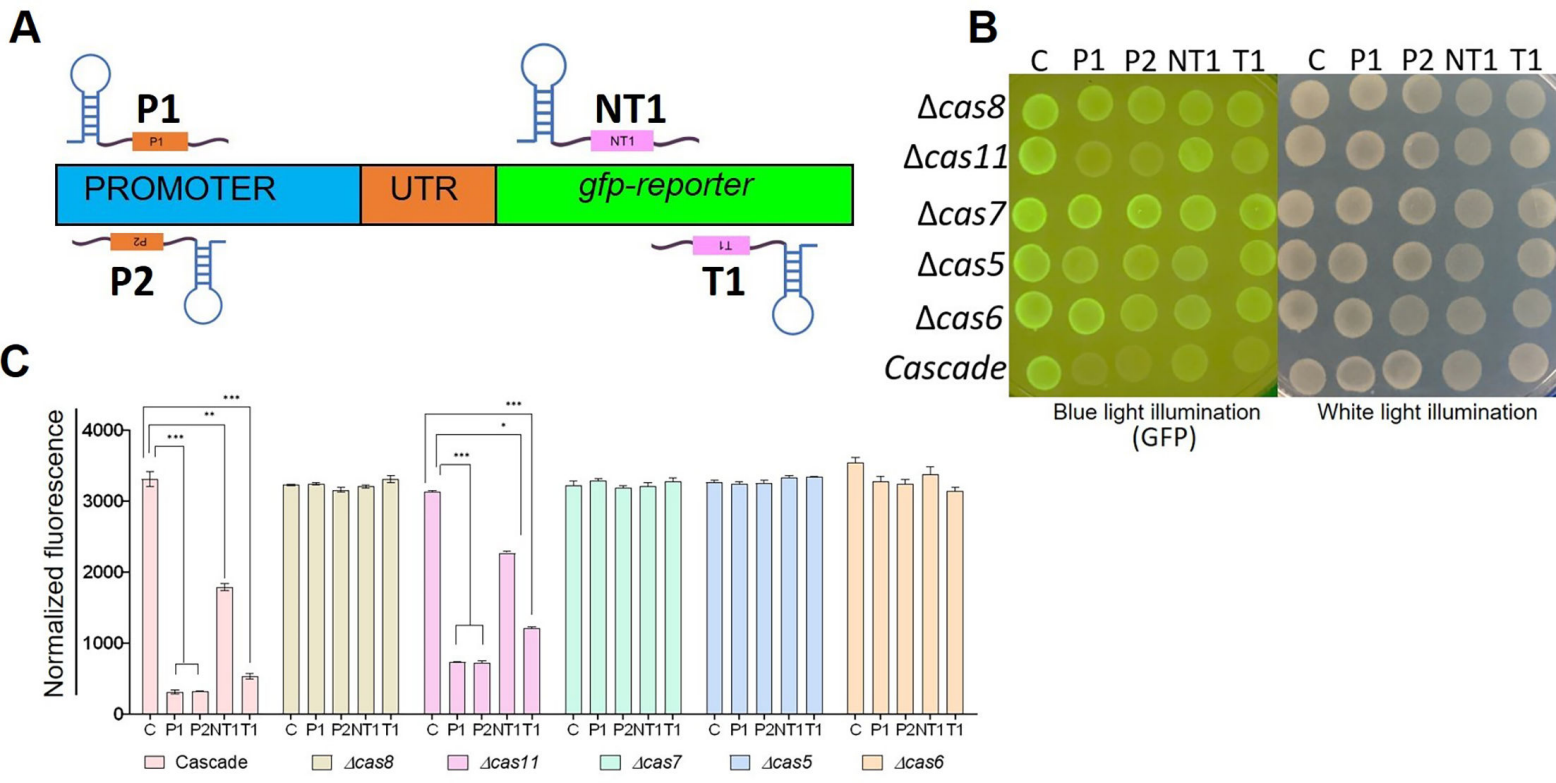
**. Effect of deletion of individual Cascade components on *gfp* silencing in *E. coli* BL21(DE3**). Binding positions of crRNAs in the promoter and the ORF of *gfp* (**A**). Fluorescence was monitored in cells expressing GFP and crRNAs (targeting *gfp* promoter, P1 and P2 or ORF, NT1 and T1 or a non-targeting scrambled control, C) and Cascade or its variants was monitored by spot assay (**B**) and in broth at 6 h post induction (**C**). Spot data are representative of experiments repeated three times (**B**). Data plotted are mean ± SEM of three independent experiments (**C**). *P < 0.05, **P < 0.01, ***P < 0.001 by *t* test.

To assess the functional essentiality of Cascade components, variants lacking one of the Cascade components were tested in gene silencing assays and compared with the full-length Cascade in *E. coli* BL21DE3 strain, a strain that lacks the genomic Cascade operon using a protocol described earlier [16]. BL21 cells carrying the *gfp* reporter under a constitutive promoter on plasmid pEH9 were transformed with plasmids expressing promoter-specific (P1, P2) or ORF-specific (NT1, T1) crRNAs (Figure 2A). Cascade or Cascade deletion variants lacking any one of the five components at a time were introduced into these strains. Gene silencing was induced, and GFP expression was monitored on agar plates, as well as broth. In cells expressing Cascade, maximum silencing was obtained with crRNA-P1 and crRNA-P2 (ten-fold), while crRNA-T1 and crRNA-NT1 could bring about six- and two-fold silencing of the reporter, respectively (Figure 2). In this genetic background, the expression of Cascade variants lacking Cas8, Cas7, Cas5 or Cas6 did not show gene silencing, suggesting that all four subunits of Cascade were essential (Figure 2B and C). Interestingly, in this strain, the Cascade variant without the Cas11 subunit displayed about half the level of gene silencing seen with complete Cascade (Figure 2B and C).

**Figure 3:**
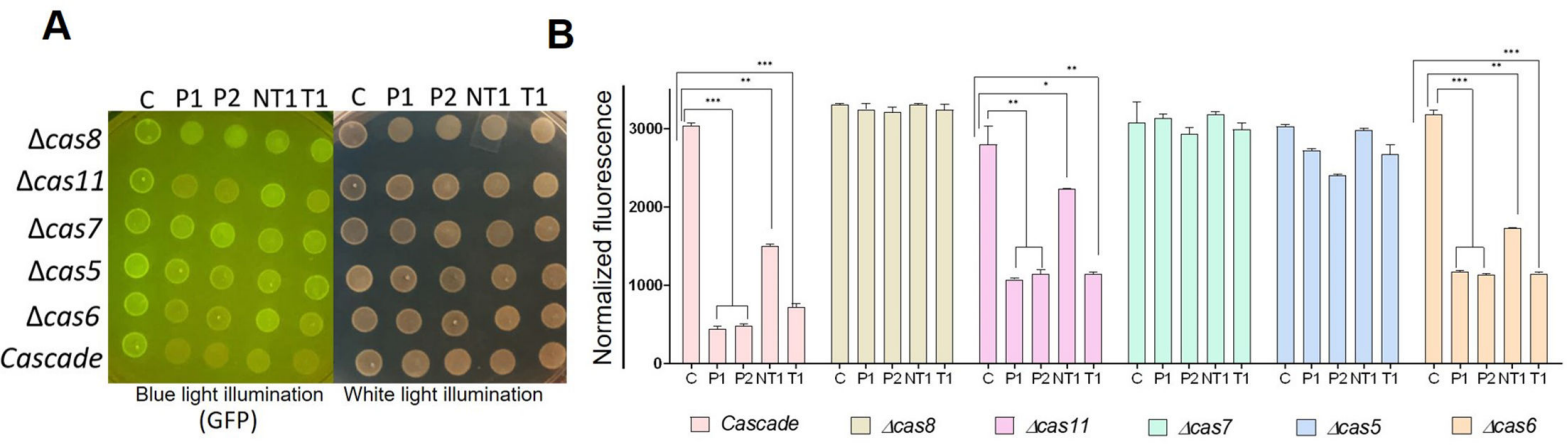
**. Effect of deletion of individual Cascade components on *gfp* silencing in *E. coli* MLS367**. Fluorescence was monitored in cells expressing GFP and crRNAs (targeting *gfp* promoter, P1 and P2 or ORF, NT1 and T1 or a non-targeting scrambled control, C) and Cascade or its variants was monitored by spot assay (**A**) and in broth at 6-h post-induction (**B**). Spot data are representative of experiments repeated three times (**A**). Data plotted are mean ± SEM of three independent experiments (**B**). *P < 0.05, **P < 0.01, ***P < 0.001 by *t* test.

The above experiments were repeated in *E. coli* MLS367 cells. In *E. coli* MLS367 cells expressing Cascade, maximum *gfp* silencing was obtained with crRNA-P1 and crRNA-P2 (6.5-fold each), followed by crRNA-T1 (4.1-fold) and crRNA-NT1 (1.9-fold) (Figure 3A and B). Deletion variants lacking *cas8*, *cas7* or *cas5* did not show silencing with any of the crRNAs, suggesting that these subunits of Cascade are essential for gene silencing. Decreased fluorescence was observed even when *cas6* or *cas11* was deleted, at about half the level of silencing obtained with Cascade (Figure 3A and B). This showed that in *E. coli* K-12 strain, Cascade lacking Cas11 or Cas6 could still give effective gene silencing, suggesting that these Cas subunits could be dispensable for gene silencing applications (Figure 3A and B). The Cascade variant without the Cas11 subunit displayed about half the level of gene silencing seen with complete Cascade (Figure 3A and B), as was observed with MLS367. While the results with Cas6 remained inconclusive, identical results in two independent genetic backgrounds show that Cas11 is dispensable for Cascade-mediated silencing of gene expression.

### Cas6 is required for Cascade-based gene silencing, but Cas11 is dispensable

**Figure 4:**
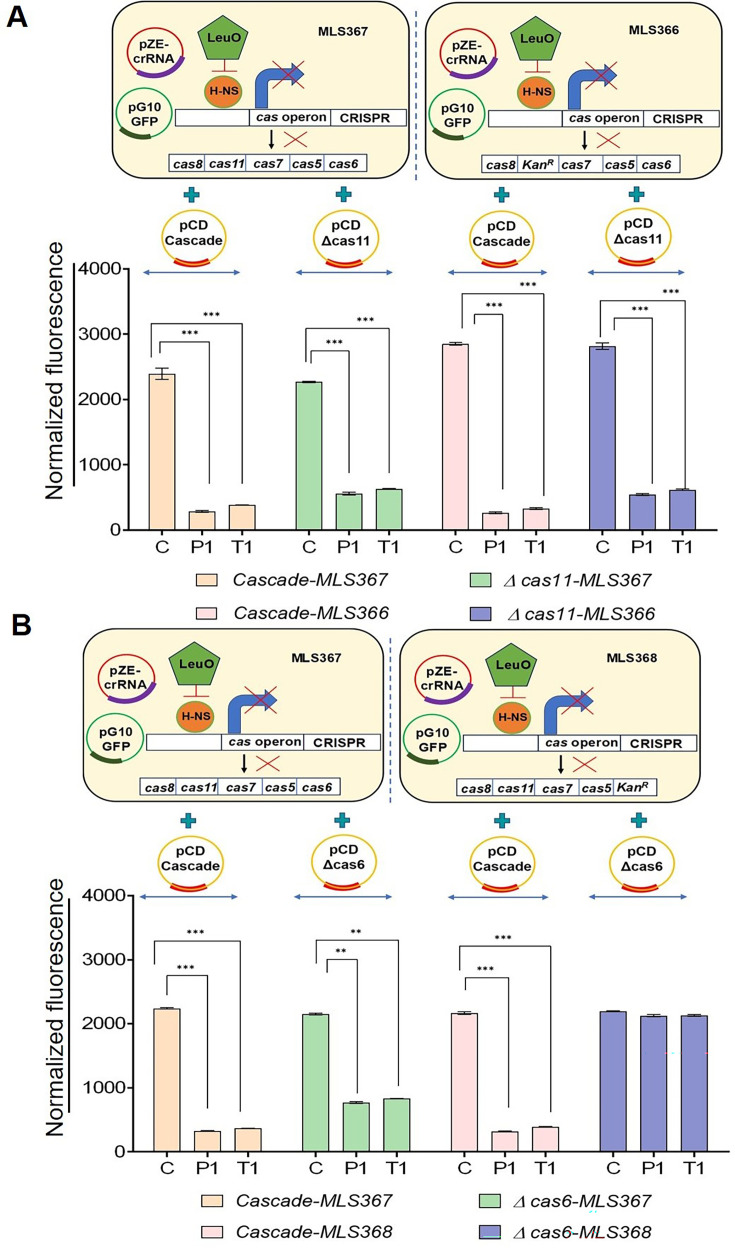
**. Effect of chromosomal deletion of cas*11* and *cas6* on *gfp* gene silencing in K-12 background of *E. coli***. Gene silencing with Cascade and ∆*cas11* Cascade expressed from plasmid in MLS367 and MLS366 (∆*cas11 MLS367*) strain (**A**). Gene silencing with Cascade and ∆*cas6* Cascade expressed from plasmid in MLS367 and MLS368 (∆*cas6 MLS367*) strain (**B)** C: non-targeting crRNA, P1 or T1: targeting crRNA. Data plotted are mean ± SEM of three independent experiments. **P < 0.01, ***P < 0.0001 by *t* test.

The observed redundancy of Cas6 for gene silencing in MLS367, a K-12 strain of *E. coli,* was intriguing. We hypothesized that it might be due to residual expression of Cas6 in an otherwise repressed Cascade operon in this strain. To ascertain this, we constructed knockouts of *cas11* and *cas6* of MLS367 by transduction using the corresponding mutants from the Keio collection [24]. Gene silencing was analyzed through in-trans complementation with plasmid-borne Cascade or its variants. Mutant cells lacking a chromosomal copy of *cas11* (MLS366) but expressing the *cas11* deletion variant of Cascade in-trans continued to show gene silencing, albeit at a lower level than obtained with complete Cascade (Figure 4A). For instance, upon using crRNA-P1, an ∼ten-fold reduction in gene silencing was observed with full-length Cascade, but a four-fold silencing was sustained upon providing Cascade lacking Cas11 (Figure 4A).

In contrast, in a strain lacking the chromosomal copy of *cas6* (MLS368), gene silencing was not observed when complemented with a Cascade lacking Cas6 (Figure 4B). However, when such cells were complemented with the full Cascade, a 6.7-fold *gfp* silencing was recorded (Figure 4B). This clearly indicated leaky expression of *cas6* from the chromosomal Cascade operon in MLS367. Together, these results suggest that while the Cas11 protein augments efficiency, it is not essential for the gene silencing function of the Cascade complex, but Cas6 is needed for its functional activity.

### Minimal Cascade lacking Cas11 can silence native gene in *E. coli*

**Figure 5:**
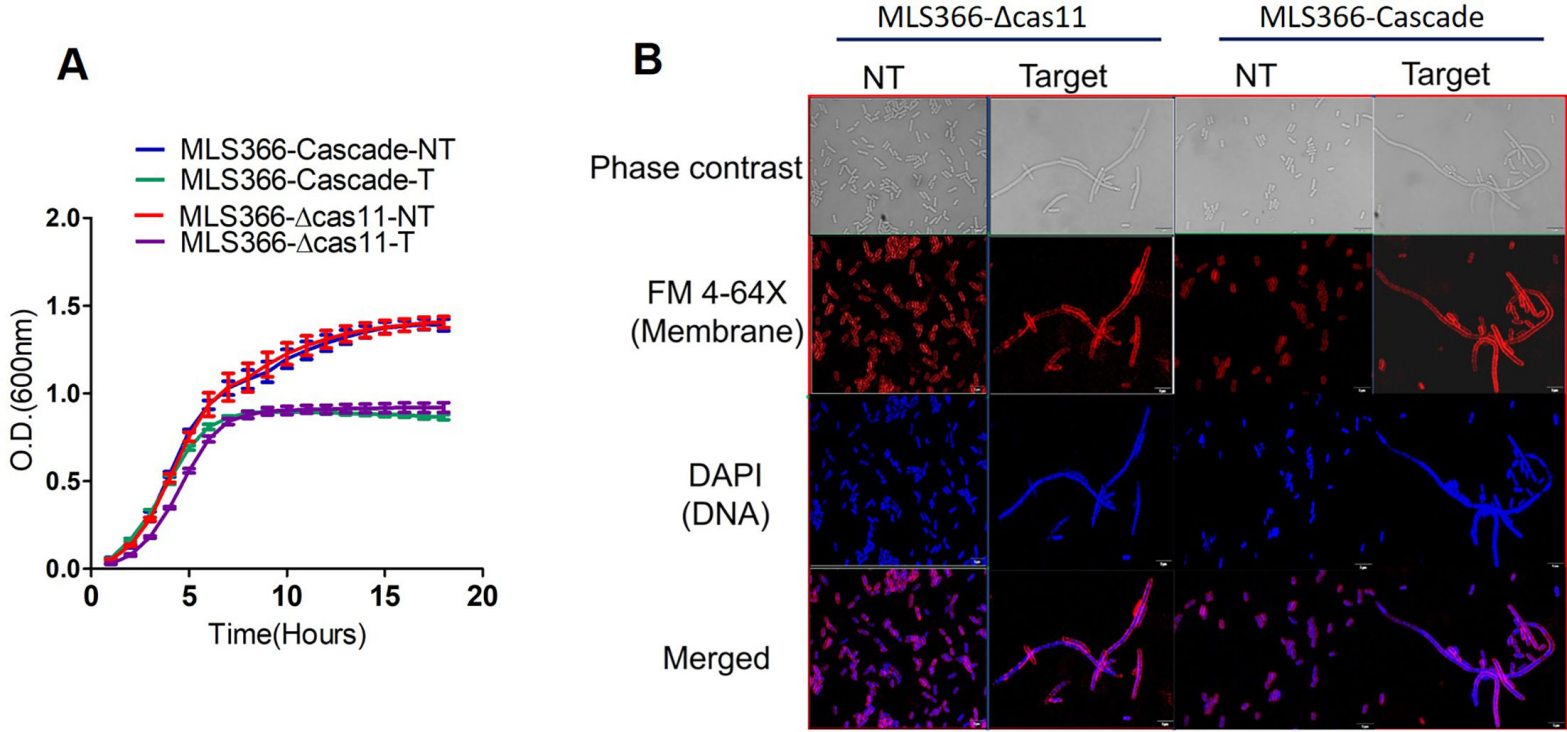
**. Effect of *racR* silencing with Cascade and *∆cas11-*Cascade**. Growth defect (**A**) and morphological defect (**B**) resulting from RacR depletion. Growth of MLS366 (*∆cas11* MLS367) cells expressing Cascade or *∆cas11* Cascade and *racR*-targeting crRNA (**T**) or non-targeting crRNA (NT) was monitored in minimal media. Cells were analyzed using confocal microscopy at 100 × magnification 5 h after the induction of transcriptional silencing. Data plotted are mean ± SEM of three independent experiments.

In order to assess and quantify gene silencing, heterologous expression of GFP from a plasmid was used as a convenient reporter in previous experiments. To show that the minimal Cascade minus the Cas11 subunit can be used to silence a native gene on the genome, we targeted *racR*. Earlier reports showed that *racR* was an essential gene within the *rac* prophage of *E. coli* K-12 strains, and its deletion made cells unviable [17]. Furthermore, Bindal et al. (2017) showed that silencing of *racR* in *E. coli* K-12 MG1655 strain significantly retarded growth and altered cell morphology, leading to the formation of long filamentous cells [17]. Silencing of *racR* in MLS366 using a targeting crRNA, either with full-length Cascade or minimal Cascade, reduced growth compared with cells expressing a nontargeting crRNA (Figure 5A). The cells were visualized by microscopy after induction of silencing. Control cells expressing a nontargeting crRNA displayed normal size and shape. However, cells expressing Cascade or Cascade lacking Cas11 and a *racR*-targeting crRNA showed striking morphological changes (Figure 5B), such as extensive filamentation and an increase in cell diameter, confirming silencing of *racR* expression in these cells. The results confirmed that the Cas11 subunit is dispensable and also that Cascade lacking this subunit can be effectively utilized for gene silencing applications.

### Cas11 is dispensable for CRISPR-Cascade-mediated plasmid interference

**Figure 6:**
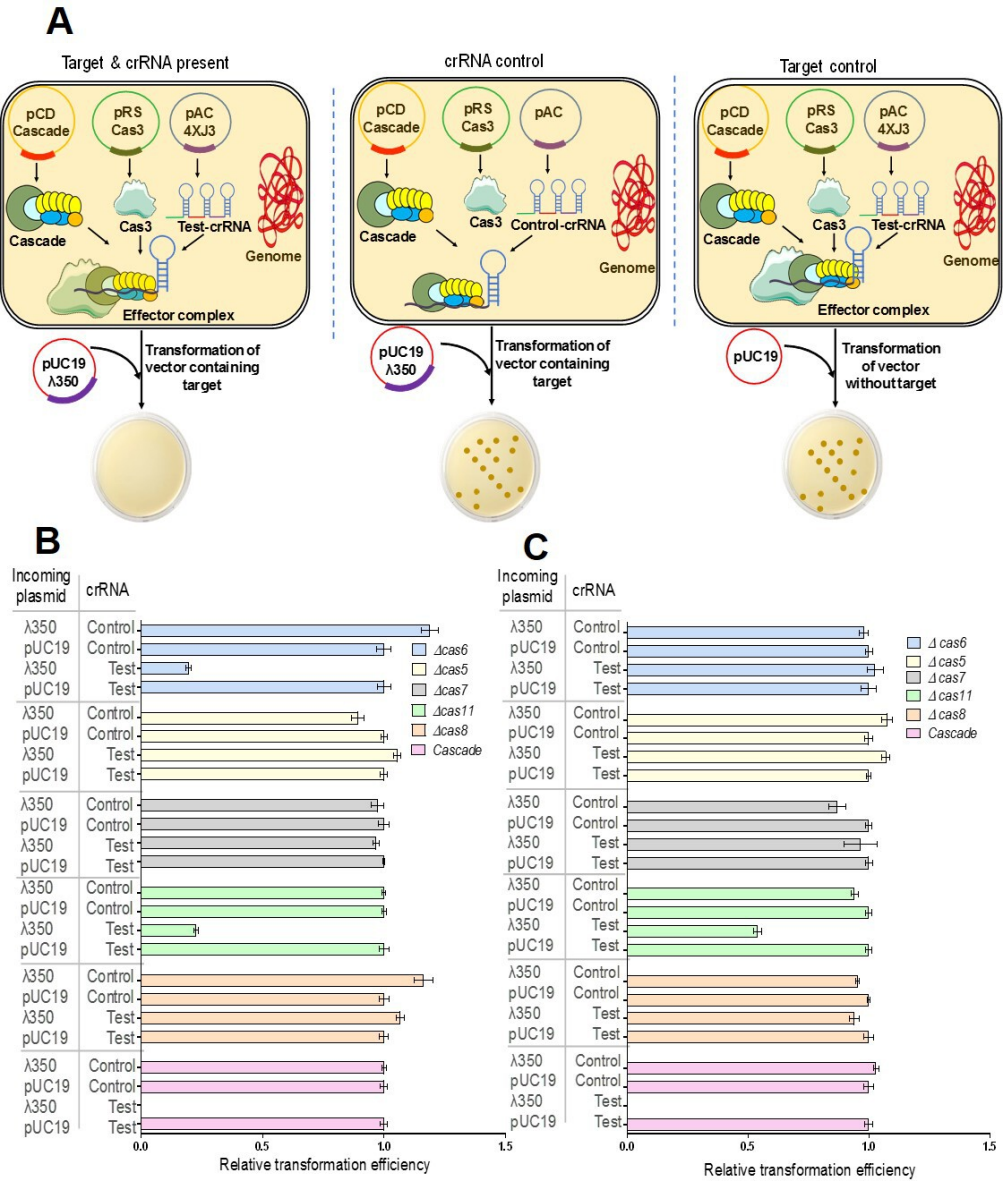
**. Plasmid interference with Cascade and Cascade variants**. A schematic for the plasmid interference assay is shown (**A**). *E. coli* MLS367 (**B**) and *E. coli* BL21AI (**C**) cells expressing Cas3, Cascade or Cascade deletion variants and targeting crRNA (Test) or a scrambled non-targeting crRNA (Control) were transformed with 100 ng of each of the plasmids, pUC19-λ350 or pUC19. Transformants recovered on ampicillin plates were enumerated. Relative transformation efficiency with respect to transformants obtained for pUC-19 control in cells expressing targeting crRNA is plotted.

Cascade is the main effector in the type IE CRISPR-Cas system that functions in bacterial immunity against mobile genetic elements (MGE). The spacer sequence of the crRNA is utilized by Cascade for recognizing the incoming MGE through RNA–DNA complementary base pairing. This leads to simultaneous recruitment of the nuclease Cas3, which eventually degrades the MGE in a processive manner, a process referred to as interference. Plasmid interference was monitored in *E. coli* cells expressing both Cas3 and Cascade or its deletion variants on plasmids. For antibiotic selection compatibility reasons, Cascade and its deletion variants used in gene silencing experiments could not be used for interference studies. Hence, Cascade or its deletion variants described earlier were sub-cloned in pCDF1b under a T7 promoter. The cells expressing Cascade deletion variants were evaluated by SDS-PAGE analysis of *E. coli*. Cells harboring different variants of Cascade in pCDF1b showed that the protein profile was similar to the profile obtained with pRSF-derivatives and broadly indicated the expression of Cascade subunits except the one deleted from the respective construct (data not shown). The stability of target plasmid pUC-λ350 (plasmid containing 350 base pairs of λ) or control plasmid pUC19 upon transformation was monitored in cells expressing Cas3, 4XJ3 (CRISPR array containing four copies of the J3 spacer targeting λ350) crRNA and Cascade or its deletion variants. The scheme depicting the experimental design of the interference assay is shown in Figure 6A. The recovery of the pUC19-λ350 (target) transformants was compared with pUC19 (non-target) transformants to determine the effect of plasmid targeting by Cascade and the deletion variants. pACYCDuet-1 carrying a crRNA against a scrambled sequence was used as a control to show that plasmid interference was dependent on the presence of targeting crRNA. The interference assays were carried out in both *E. coli* K-12 (MLS367) and B strain (BL21) backgrounds (Figure 6B and C). Results showed that transformants for pUC-λ350 could not be recovered in the presence of full-length Cascade and the targeting 4XJ3 test crRNA in both *E. coli* K-12 (BW25113) and B strain (BL21) backgrounds (Figure 6B and C), while good transformation efficiency was obtained with pUC19 vector alone. However, the number of transformants recovered was similar upon transforming pUC19 or pUC-λ350 in cells expressing a scrambled non-targeting control crRNA. This showed that the plasmid interference was equally functional in both the genetic backgrounds. With Cascade variants, results were similar to those obtained with gene silencing assays. Plasmid interference did not take place upon deletion of *cas8, cas7* or *cas5* in either of the backgrounds, suggesting that these three subunits of Cascade are essential for interference. Cascade without Cas6 affected plasmid stability in K-12 background (Figure 6B), but this was not observed in B strain background (Figure 6C). Interestingly, both strains of *E. coli* showed low plasmid stability in the presence of minimal Cascade lacking Cas11, suggesting that Cascade lacking Cas11 could still support interference. In BW25113, there was a four-fold reduction in the number of transformants in the absence of Cas11, when the cells are transformed with the target plasmid, whereas in BL21, there was only a two-fold reduction. In summary, these results demonstrate that the Cascade complex effectively targets and eliminates plasmids in the K-12 background, even in the absence of Cas11 or Cas6. However, in the B strain background, Cas6 is essential, while Cas11 is dispensable. Experiments to confirm the dispensability of *cas6* in the K-12 strain could not be performed due to the inability to generate a mutant with an antibiotic cassette insertion that was unique from those being engaged for selection of the rest of the four plasmids required for interference.

### Discussion

The sea-horse–shaped structure of the type I-E CRISPR system from *E. coli* has been studied in detail and characterized extensively [25-27]. The function of each of the subunits is well annotated and experimentally verified. The crystal structure of the *E. coli* Cascade complex, resolved at 3.05 Å, showed that six Cas7 proteins, along with Cas5 and Cas6, create a tightly packed outer layer, while the inner layer consists of a Cas8 and Cas11 dimer. The structure revealed that the 61-nucleotide crRNA spans the entire complex, interacting with all six Cas7 subunits. The inner layer connects to the outer layer primarily through Cas8–Cas5 interactions, supplemented by multiple, relatively weak contact points between Cas7 and the lateral regions of the Cas11 dimer that occupies the ‘belly’ region on the Cascade complex [9].

There has been a plethora of *in vitro* studies on Cascade components and their interaction with crRNA and/or target DNA. Early studies found that the absence of Cas8 and Cas11 did not affect the structural stability of the remaining subunits in the complex, nor did it interfere with the formation of mature crRNA [8]. A recent study demonstrated, *in vitro*, that the Cas5-Cas7 scaffold is sufficient for PAM-independent DNA targeting when using a mature crRNA. The study also showed R-loop formation and DNA cleavage in the absence of Cas11 but at much lower efficiency compared with full-length Cascade [28].

There have been few studies *in vivo* that have looked at minimal Cascade in the type I-E system. This is perhaps the first study to assess gene silencing with Cascade variants. Our work shows that Cas11 is dispensable not only for Cascade-mediated gene silencing that involves binding of the surveillance complex to a specific DNA molecule but also for plasmid interference, which involves, in addition to DNA binding, Cas3 recruitment *in vivo* and DNA cleavage. Jore et al. expressed single-component deletion variants of Cascade from plasmid in BL21(DE3), which is identical with our study, and found no lambda phage interference in any of the variants, concluding that all components are essential [8]. Our results are in variance, and we show that plasmid interference is supported by the Cas11 deletion variant of Cascade. These apparently different outcomes could be due to different genetic elements used for expression of type I-E CRISPR system components or differences in the requirement for plasmid vs. phage interference.

Structure-based studies have shown that Cas11 interacts with the non-target strand and locks it. This is hypothesized to aid stabilization of the R-loop [26] and consequent conformational change in the Cascade complex to enable Cas3 recruitment for target cleavage. We hypothesized that as interference requires additional steps over CRISPRi, such as recruitment of Cas3, stabilization of R-loop and activation of nuclease, the requirement of Cas components could be different for DNA cleavage or at least more stringent than CRISPRi. Dispensability of Cas11 for DNA cleavage shown in our study indicates that the role of Cas11 in stabilization of R-loop may not be crucial for Cas3-mediated cleavage *in vivo*. The melting of the DNA strand guides the non-target strand away from the target to stabilize the R-loop and maybe sufficient for Cas3 recruitment, with the locking by Cas11 providing extra stability to this structure preventing R-loop collapse [26]. Accordingly, in our studies, though deletion of Cas11 could still bring about interference, the efficiency was compromised, perhaps due to an unstable R-loop structure affecting Cas3 recruitment. This also appears consistent with the *in vitro* study [28]. Furthermore, another study has shown a fair degree of flexibility in the association of Cascade subunits with crRNA and with each other (29). This flexibility could also come into play in *in vivo* conditions, where overexpression of other Cascade components may compensate for the absence of Cas11 to provide structural stability, supporting the notion of a dynamically adaptable Cascade architecture.

While Cascade type I-E encodes a distinct Cas11 subunit, the presence of Cas11 in type I-B, I-C and I-D went unnoticed until recently. The type I-D Cascade from *Synechocystis* sp. PCC 6803 had an alternative internal translational initiation site within *cas10d* that led to the expression of the small subunit, Cas11d [30]. DNA binding was drastically reduced in the absence of the Cas11d subunit. The minimal type 1-C system from *Neisseria lactamica* had a ‘hidden’ *cas11* sequence within the *cas8* sequence and was required for genome editing in eukaryotes [31]. Type I-C system from *Desulfovibrio vulgaris* similarly had a ‘hidden’ *cas11,* which was a strict requirement for successful genome editing [32]. The type I-B system from *Synechocystis* also carries a hidden *cas11* that significantly boosted editing efficiency when supplied separately [31]. The type I-F CRISPR system, on the other hand, lacks Cas11. Protection against lambda phage was demonstrated using Type 1-Fv of this minimal version [33]. The dispensability of Cas11 for a functional Cascade, therefore, appears to vary for each subtype of Cascade.

We also demonstrated that the minimal I-E CRISPR-Cas system from *E. coli* can effectively silence *gfp* gene expression and interfere with plasmid transformation, even in the absence of Cas6 activity in the BW5113 strain. In natural conditions, the expression of the Cascade operon in *E. coli* is known to be repressed in this strain [23,34]. However, the indispensability of Cas6 in the BL21 strain, which is a natural mutant for the Cascade locus, shows that the Cascade complex indeed needs this subunit for DNA binding and cleavage when a pre-crRNA is provided. The dispensability of Cas6 in the BW5113 strain is puzzling, especially since any background expression was ruled out by checking for silencing of the *gfp* gene from the chromosomal Cascade alleles. One possibility could be that the Cascade locus is expressed from the chromosome at very low levels in BW5113 that are insufficient for the formation of an active Cascade complex. Since Cas6 is employed only in a single copy in the Cascade complex, this level of expression might have been sufficient to engage with the rest of Cascade subunits expressed in excess from the plasmid to bring about *gfp* silencing. Why this was not true for Cas8 and Cas5, which are also engaged in single copy in the Cascade complex, is not quite clear. Cas6, a component of the Cascade complex and an endoribonuclease, typically generates unit-sized crRNA from the pre-crRNA precursor transcript and is essential for CRISPR-Cas function. It was also shown that when mature unit-sized crRNAs are provided through transcription termination, the Cascade complex could bring about bacteriophage interference even in the absence of Cas6 in *E. coli* [12]. In the type I-B system from *Haloferax* also, it has been shown that Cas6 is not essential for crRNA binding to the Cascade complex or for target recognition [35]. Removal of all but one nucleotide of the 3′ handle of crRNA was shown to still bind type 1 Cascade *in vitro* [36] as well as *in vivo* [35]. Furthermore, Cascade lacking Cas6 could still recognize the target when provided with a mature crRNA lacking the 3′ handle *in vitro* [12]. Our study has shown the dispensability of the Cas11 protein when a full pre-crRNA was provided for DNA binding and cleavage *in vivo*. It remains to be seen if a minimal Cascade without Cas6 and Cas11 can still form a stable complex with a mature crRNA lacking the 3′ handle for application in genome editing and silencing.

## Materials and methods

### Strains and culture media

Most experiments were performed in the *E. coli* strain MLS367, which is a Δcas3 derivative of E. coli K-12 BW25113 with an arabinose-inducible T7 polymerase (ara::T7RNAP-tetA) gene integrated into its genome [16] and BL21DE3 or BL21AI strain that naturally lack Cas3 and Cascade. *E. coli* was cultured in Luria Bertani (LB) medium or minimal media [17] and grown with aeration at 37°C. The media was supplemented with kanamycin (25 μg/ml), ampicillin (100 μg/ml) and chloramphenicol (15 μg/ml), wherever required. To construct *cas11* and *cas6* mutants of MLS367, Keio collection [24] strains JW2726 (Δ*cas11::kan*) and JW2729 (Δ*cas6::kan*) were used as donors in P1 transduction [37]. MLS367 was used as a recipient, and recombinants were selected on kanamycin. Δcas11 and Δ*cas6* derivatives of MLS367 were named as MLS366 and MLS368, respectively. Details of *E. coli* strains used in the present study are given in Supplementary Table S1.

### Construction of recombinant plasmids

For recombinant plasmid construction and transformation, standard methods as described were used [38]. The recombinants were confirmed by restriction analysis and sequencing as required. The Cascade operon was cloned in pRSF1b or pCDF1b at the NcoI and NotI sites, and the resulting plasmids were named pRS-Cas or pCD-Cas, respectively. Various deletion variants of the Cascade operon were generated from these two recombinants by designing inverse primers for the targeted deletion of individual genes. As an example, for the deletion of *cas8* (pRS-Δcas8), the inverse primers binding upstream and downstream of *cas8* were used for amplification, and the product was self-ligated to generate pRS-Δcas8. Similarly, for the generation of pRS-Δcas11, pRS-Δcas7, pRS-Δcas5 and pRS-Δcas6, primers binding upstream and downstream of the respective ORFs were used for inverse PCR and self-ligation of the products. The deletion of individual Cas genes was confirmed by diagnostic PCR using gene-specific and vector-specific primers and restriction enzyme digestion analysis of resultant plasmids. For expression of crRNA, CRISPR arrays containing the transcribed 53 bp of the leader sequence from the CRISPR1 array of *E. coli* K-12 MG1655 and a spacer flanked by two repeats were synthesized and cloned into the EcoRI and XbaI sites of pZE12Luc as reported earlier [16]. For gene silencing experiments, MLS367, BL21DE3, MLS368 and MLS366 cultures were serially transformed with pEH9 carrying *gfp*, crRNA-expressing plasmids and pRS-Cascade or its deletion variants (pRS-Δcas6, pRS-Δcas5, pRS-Δcas7, pRS-Δcas11 and pRS-Δcas8). Plasmids used in the present study are listed in Supplementary Table S2. Primers used in the present study are given in Supplementary Table S3.

### Protein expression and analysis

The Cascade complex and its deletion variants were expressed in *E. coli* BL21 (DE3) in LB broth with kanamycin (25 µg/ml) or streptomycin (15 µg/ml). After growing to optical density (OD) 600 nm ∼0.5 at 37°C, expression was induced with 0.5 mM IPTG for 3 h. Cells were harvested, lysed and debris removed by centrifugation. The proteins were separated on SDS-PAGE gel and stained using Coomassie Brilliant Blue.

### Gene silencing studies

To evaluate the extent of *gfp* silencing in the *E. coli* population, cultures grown overnight in LB medium were diluted 1:100 into 20 mL of fresh LB medium containing the appropriate antibiotics, 0.2% arabinose and 0.5 mM IPTG to induce the expression of crRNA and Cascade or its variants. The cultures were then incubated with aeration in 100-mL flasks at 37°C. OD600 and fluorescence at 520 nm were measured at 5-h post-inoculation during the exponential phase. For fluorescence measurements, 200 μl aliquots of the cultures were transferred to black Corning flat-bottom plates and read using an Infinite M200 PRO microplate reader (Tecan), (Excitation λ = 480 nm, Emission λ = 520 nm). Fluorescence was normalized to OD values to account for variations in cell density. This normalization allowed for the assessment of relative fluorescence intensity.

### Plasmid interference studies

A 350-bp fragment derived from the J gene of λ phage (λ350) cloned in pUC19 served as target for plasmid interference. *E. coli* (MLS367/BL21) cells, expressing crRNA 4XJ3 (targeting λ350) from pACYCduet1 plasmid, Cas3 from pRSF1b plasmid and Cascade, or its deletion variants on pCDF1b plasmid served as host. Cells expressing Cascade or its variants, crRNA and Cas3 were transformed with 100 ng each of an empty vector control plasmid, pUC-19 or pUC19-λ350 test plasmid. Electroporation was done at 1400 volts for high efficiency of transformation using the Electroporator 2510 Eppendorf instrument. The cells were plated on LB-agar plates containing inducers, IPTG and arabinose for expression of the Cas3, Cascade and crRNA and ampicillin for selection of pUC19/pUC19- λ350 transformants. After overnight incubation, plasmid stability was evaluated by comparing the number of colony-forming units on the control plates to those on the test plates.

### Microscopy

The cells containing the silencing machinery were harvested after 5 h of induction and washed twice with 1 × phosphate-buffered saline (PBS). For fluorescence microscopy, the cells were stained with 4′,6′-diamidino-2-phenylindole (0.5 μg/μl) to label the nuclei and FM 4–64FX (0.5 μg/μl), a membrane stain, for 30 min at room temperature in the dark. After staining, the cells were washed three times with 1 × PBS and mounted onto a 1% agarose bed on a glass slide. Images were captured using a confocal microscope (Olympus IX83 FluoView 3000) with 100 × magnification. The images were processed using cellSens imaging software, and representative images have been shown.

## Supplementary material

Online supplementary material 1

## Data Availability

Data will be made available by corresponding author at reasonable request.

## Abbreviations

CRISPR: Clustered Regularly Interspaced Short Palindromic Repeats
IPTG: Isopropyl β-D-thiogalactopyranoside
LB: Luria Bertani
MGE: mobile genetic elements
OD: Optical density
PAM: protospacer-adjacent motif
