# Supplementary material for: Cas11 augments Cascade functions in type I-E CRISPR system but is redundant for gene silencing and plasmid interference
Source: Biochem J. 2025 Jun 11;482(12):793–805. doi: 10.1042/BCJ20253056 (PMC12235044; doi:10.1042/BCJ20253056)
Supplement: Online supplementary material 1 [file bcj-482-12-BCJ20253056-supp1.docx]

**Supporting Information**

**Cas11 augments Cascade functions in type I-E CRISPR system but is redundant for gene silencing and plasmid interference**

Neha Pandey ^a b^, Chitra S. Misra ^a^, Devashish Rath ^a c *^

^a^ Applied Genomics Section, Bio-Science Group, Bhabha Atomic Research Centre, Mumbai, India 400085.

^b^ Life Sciences, Mumbai University, Vidya Nagari, Kalina, Santacruz East, Mumbai, India 400098.

^c^ Homi Bhabha National Institute, Anushaktinagar, Mumbai, India 400094.

* Correspondence: Devashish Rath, Email: [devrath@barc.gov.in](mailto:devrath@barc.gov.in) phone: +91 22 25590796

**Contents:**

**Supporting Figures**

**Fig. S1**

**Fig. S2**

**Supporting Tables**

**Table S1**

**Table S2**

**Table S3**

**Fig. S1. PCR analysis to confirm presence/absence of type I CRISPR system in *E. coli* strains.** (A) Amplification of Cascade operon from the genomic DNA. (B) Amplification of *cas3* from the genomic DNA.


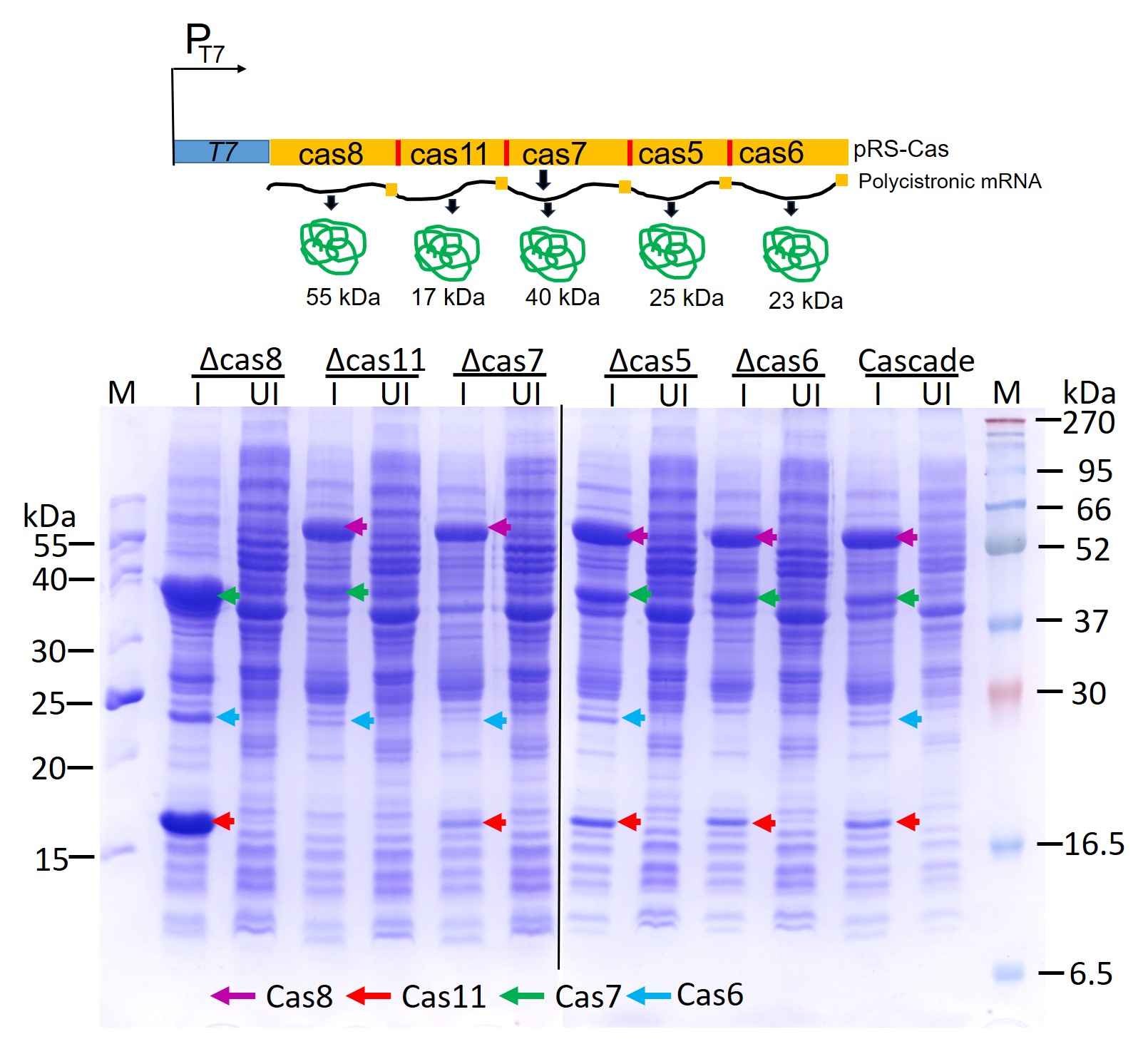


**Fig. S2. SDS-PAGE Analysis of Cascade deletion variants.** The Cascade operon expresses Cas proteins of various molecular weights (A). Coomassie Brilliant Blue stained SDS-PAGE gel showing proteins extracted from BL21(DE3) cells expressing Cascade or its deletion variants in absence (UI) or presence (I) of inducer (B). The results highlight the loss of expression of deleted genes and the overexpression of proximal genes, with arrows indicating the expression of different Cas proteins in the deletion variants.

**Table S1: List of strains**

| **Strains** | **Description** | **Source or reference** |
| --- | --- | --- |
| *Escherichia coli* K-12 BW25113 | F^-^ λ^-^ *rrnB3* Δ*lacZ4787* *hsdR514* Δ(*araBAD*)567  Δ(*rhaBAD*)568 *rph-1* | Baba et al. (2006) |
| *E. coli* DH5α | F^-^ λ^-^ *endA1* *glnV44 thi-1 recA1 relA1 gyrA96 deoR* *nupG* *purB20* Φ80d *lacZ*ΔM15 Δ(*lacZYA-argF*)U169, *hsdR17*(r_K_- m_K_+) | Lab collection |
| *E. coli* BL21(DE3) | F^-^ *ompT gal dcm lon hsdS_B_*(r_B_- m_B_-) λ(DE3 [*lacI lacUV5*-T7p07 *ind1* *sam7* *nin5*]) [*malB+]_K-12_(*λ^S^*)* | Lab collection |
| *E. coli* BL21(AI) | F^-^ *ompT gal dcm lon hsdS_B_*( r_B_- m_B_-)  [*malB+]_K-12_(*λ^S^*) araB::T7RNAP-tetA* | Lab collection |
| *E. coli* K-12 MLS367 | BW25113 Δ*cas3* *araB*::*T7RNAP-tetA* | Rath et al. (2015) |
| *E. coli* K-12 JW2729 | BW25113 Δ*cas11::Kan* | Baba et al. (2006) |
| *E. coli* K-12 JW2726 | BW25113 Δ*cas6::Kan* | Baba et al. (2006) |
| *E. coli* K-12 MLS366 | As MLS367 transduced with Δ*cas11::Kan* | This study |
| *E. coli* K-12 MLS368 | As MLS367 transduced with Δ*cas6::Kan* | This study |

**Table S2: List of plasmids**

| **Plasmid** | **Description** | **Restriction**  **sites** | **Source/Ref** |
| --- | --- | --- | --- |
| pRSF-1b | T7 RNA polymerase based expression vector, Kan^R^ |  | a |
| pCDF-1b | T7 RNA polymerase based expression vector, Strep^R^ |  | a |
| pACYC-duet1 | T7 RNA polymerase based expression vector, Cam^R^ |  | a |
| pZe12luc | Vector for *E. coli* CRISPR array expression |  | a |
| pZE12luc-crRNA1-C | Source of non-targeting control spacer |  | Rath et al. (2015) |
| pZE12luc-crRNA2- P1 | Source of promoter targeting spacer |  | Rath et al. (2015) |
| pZE12luc-crRNA3-P2 | Source of promoter targeting spacer |  | Rath et al. (2015) |
| pZE12luc-crRNA4-NT1 | Source of ORF targeting spacer |  | Rath et al. (2015) |
| pZE12luc-crRNA5-T1 | Source of ORF targeting spacer |  | Rath et al. (2015) |
| pEH9 | *gfp* reporter plasmid |  | Rath et al. (2015) |
| pWUR400 | Cascade cloned in pCDF-1b |  | Brouns et al. (2008) |
| pWUR397 | *cas3* in pRSF-1b, no tags |  | Brouns et al. (2008) |
| pZE12luc-T | Source of targeting spacer for *racR* |  | Bindal et al. (2017) |
| pZE12luc-NT | Source of non-targeting spacer for *racR* |  | Bindal et al. (2017) |
| pAC-4XJ3 | 4XJ3 spacer in pACYC-duet1 | BamHI/XhoI | This study |
| pUC19 | Cloning Plasmid |  | Lab stock |
| pUC-λ350 | λ350 in pUC19 | BamHI/EcoRI | This study |
| pRS-cascade | *cas8-cas11-cas7-cas5-cas6* in pRSF-1b, no tags | NcoI/NotI | This study |
| pRS-Δcas6 | *cas8-cas11-cas7-cas5* in pRSF-1b, no tags | NcoI/NotI | This study |
| pRS-Δcas5 | *cas8-cas11-cas7-cas6* in pRSF-1b, no tags | SalI | This study |
| pRS-Δcas7 | *cas8-cas11-cas5-cas6* in pRSF-1b, no tags | SalI | This study |
| pRS-Δcas11 | *cas8-cas7-cas5-cas6e* in pRSF-1b, no tags | SalI | This study |
| pRS-Δcas8 | *cas11-cas7-cas5-cas6e* in pRSF-1b, no tags | NcoI | This study |
| pCD-Δcas6 | *cas8-cas11-cas7-cas5* in pCDF-1b, no tags | NcoI/NotI | This study |
| pCD-Δcas5 | *cas8-cas11-cas7-cas6* in pCDF-1b, no tags | NcoI/NotI | This study |
| pCD-Δcas7 | *cas8-cas11-cas5-cas6* in pCDF-1b, no tags | SalI | This study |
| pCD-Δcas11 | *cas11-cas7-cas5-cas6* in pCDF-1b, no tags | SalI | This study |
| pCD-Δcas8 | *cas11-cas7-cas5-cas6* in pCDF-1b, no tags | NcoI | This study |

^a^ pRSF-1b, pCDF-1b, pACYC-duet1, pZE12luc, pWUR400 and pWUR397 were a kind gift from Magnus Lundgren, Uppsala University.

**Table S3: List of primers**

| **Primer** | **Sequence (5’-3’)** | **Description** |
| --- | --- | --- |
| NP1 | GTCCATGGATGGCTAATTTGCTTATTGATAACTGGATCC | For cloning of Cascade |
| NP2 | GGAGCGGCCGCTATCTCAGTAAAGTCATCATTGCC |  |
| NP1 | GTCCATGGATGGCTAATTTGCTTATTGATAACTGGATCC | For deletion of *cas6* from pRS-cascade |
| NP3 | GGAGCGGCCGCCTGAGATACATCCATACCTCCTTTAATC |  |
| NP4 | gtagtcgactcacgcctcgccattattacgaac | For deletion of *cas5* from pRS-cascade |
| NP5 | gtagtcgacgattaaaggaggtatggatgtatctcagtaaagtc |  |
| NP6 | GTAGTCGACtggcgaggcgtgaacatgagatc | For deletion of *cas7* from pRS-cascade |
| NP7 | GTAGTCGActtacgcatttttgtttgtggtcaatac |  |
| NP8 | GTAGTCGACaggaaacctttctatgtctaacTTTATC | For deletion of *cas11* from pRS-cascade |
| NP9 | GTAGTCGACTCAgccatttgatggccctCC |  |
| NP10 | gtaccatggcTGAtgaaattgatGCaatg | For deletion of *cas8* from pRS-cascade |
| NP11 | gtagccatgGTATATCTCCTTATTAAAGTTAAAC |  |
| NP12 | GGACATATGTTAATAATAAGGAAATGTTACATTAAGG | For cloning of 4XJ3 spacer |
| NP13 | GGACTCGAGGGGTTTGAAAATGGGAG |  |
| NP14 | ATAGGATCCTCGGGCGAGCGATGATGCG | For cloning of λ350 fragment |
| NP15 | TACTGGAATTCCATCGGCGTTTCATTCCCGTTT |  |
| P1 | GGAATTCATATGGCTAATTTGCTTATTGATAACTGGATCC | For screening of  Δcas11 mutant strain(MLS366) |
| P2 | CCGCTCGAGCGCCTCGCCATTATTACGAACCC |  |
| P3 | GGAATTCATATGAGATCTTATTTGATCTTGCGGC | For screening of  Δcas6 mutant strain(MLS368) |
| P4 | CCGCTCGAGTCAGCTACTCCGATGGCCTGC |  |
| Cas6R | CCGCTCGAGcacagtggagccaaagatagc | For screening of  pRS-Δcas5 recombinants |
| T7F | TAATACGACTCACTATAGGG | For screening of  Cascade and its deletion variants |
| T7R | GCTAGTTATTGCTCAGCGG | For screening of  pRS-Δcas6 recombinants |
| Cas3F1 | gatcctcgagatggaaccttttaaatatatatgccat | For *cas3* amplification |
| Cas3R1 | tactggaattcttatttgggatttgcagggatgactctggtc | For *cas3* amplification |

**References**

Baba T, Ara T, Hasegawa M, Takai Y, Okumura Y, Baba M, Datsenko KA, Tomita M, Wanner BL, Mori H. Construction of Escherichia coli K-12 in-frame, single-gene knockout mutants: the Keio collection. Mol Syst Biol. 2006;2:2006.0008.

Rath D, Amlinger L, Hoekzema M, Devulapally PR, Lundgren M. 2015. Efficient programmable gene silencing by Cascade. Nucleic Acids Res 43:237–246

Brouns SJJ, Jore MM, Lundgren M, Westra ER, Slijkhuis RJH, Snijders APL, Dickman MJ,Makarova KS, Koonin EV, van der Oost J. 2008. Small CRISPR RNAs guide antiviral defense in prokaryotes. Science 321:960–964.

Bindal G, Krishnamurthi R, Seshasayee ASN, Rath D. CRISPR-Cas-Mediated Gene Silencing Reveals RacR To Be a Negative Regulator of YdaS and YdaT Toxins in *Escherichia coli* K-12. mSphere. 2017 Dec 27;2(6).
